# Acid Precipitation and the Prevalence of Parkinson’s Disease: An Ecologic Study in U.S. States

**DOI:** 10.3390/brainsci11060779

**Published:** 2021-06-12

**Authors:** Gary G. Schwartz, Mark R. Williamson

**Affiliations:** Department of Population Health, School of Medicine & Health Sciences, University of North Dakota, Grand Forks, ND 58203, USA; Mark.williamson.2@und.edu

**Keywords:** epidemiology, Parkinson’s disease, acid rain, etiology, hypothesis

## Abstract

Although the etiology of Parkinson’s disease (PD) is unknown, potentially informative clues lie in its geographic distribution. PD prevalence rates within the U.S. are significantly higher in the Midwest and Northeast, a pattern that resembles the geographic distribution of acid precipitation (“acid rain”). Using linear and multivariable regression, we examined state-wide data on PD prevalence in relation to environmental factors including total precipitation, the acidity of precipitation, the use of well water, and industrial releases of sulfuric acid. In multivariate analyses, age-, race-, and gender-adjusted prevalence rates for PD were inversely correlated with well water use and positively correlated with industrial releases of sulfuric acid and with the quantity of acid precipitation (*p* < 0.0001). To our knowledge, this is the first report of an association between PD and acid rain. Because acid rain is known to leach metals from soils and pipes into drinking water, acid rain’s association with PD prevalence adds support for a role for metals in the etiology of PD.

## 1. Introduction

With more than 6 million affected persons in 2016, Parkinson’s disease (PD) is the second most common neurodegenerative disease. The incidence and prevalence of PD worldwide has increased markedly in the past 20 years. The increase is greatest in countries that have experienced rapid modernization, suggesting that PD may be related to some by-product of industrialization [1,2].

PD is characterized pathologically by the loss of dopamine-producing neurons in the substantia nigra and by the presence of intracytoplasmic inclusions (Lewy bodies) that contain α-synuclein. Approximately 5–15% of PD cases are familial [3]. The remaining 85–95% are sporadic (idiopathic), and their causes are believed to be environmental. Established risk factors for idiopathic PD include exposure to the synthetic toxin MPTP and to some pesticides [4]. Other possible risk factors include exposure to solvents, heavy metals, and well water [5]. However, the cause of most PD cases is unknown.

Clues to the etiology of idiopathic PD may lie in its geography. For example, PD prevalence rates within the U.S. are significantly higher in the Midwest and Northeast [6]. This pattern is reminiscent of the geography of acid precipitation (aka “acid rain” and “acid deposition”) [7,8]. Acid rain, i.e., precipitation with a pH < 5.6, results when sulfur and nitrogen compounds mix in the atmosphere. The principal sources of these compounds are coal-burning power plants, automobile exhausts, and industrial chemical releases [9].

Acid precipitation could play a role in the etiology of PD via the leaching of metals that are present in soil and in water reservoirs into drinking water [10]. This study examined the association of acid precipitation and other environmental factors with PD prevalence rates in U.S. states. We report that PD prevalence rates are highly correlated with measures of acid rain.

## 2. Materials and Methods

### 2.1. PD Prevalence

Data on PD prevalence in U.S. states were obtained from Mantri and colleagues [11], who calculated age-, race-, and sex-adjusted PD prevalence rates using a population-based sample of >27,000,000 Medicare beneficiaries [11].

### 2.2. Exposure Variables

#### 2.2.1. Annual Precipitation

We used data from publicly available sources on the average quantity of precipitation per state [12].

#### 2.2.2. pH of Precipitation

Data on the pH of precipitation were obtained from the National Atmospheric Deposition Program [13]. The dataset included pH concentrations across various U.S. sites for multiple years. We calculated the mean pH per state for the year 1994. We selected 1994 because of the long latency period of PD and because maps of acid rain for that year were available via the Encyclopedia Britannica (https://www.britannica.com/science/acid-rain/History, accessed on 25 March 2021).

The pH scale goes from 0 to 14, with values < 7 (neutral) being acidic and values > 7 alkaline (basic). The scale is logarithmic; a change in one unit reflects a 10-fold difference in acidity/alkalinity.

#### 2.2.3. Acid Precipitation Index

We reasoned that the effects of acid precipitation would depend upon the acidity of the precipitation and its quantity. We created a variable, the “Acid Precipitation Index” (API), to reflect this. To form the API, we subtracted a state’s mean pH from neutral pH (i.e., 7) and multiplied that quantity by the state’s annual precipitation (i.e., (7-observed pH of precipitation of State “A”) × (annual precipitation of State ”A”)).

#### 2.2.4. Well Water Use

Data on the percentage of public water use by state were obtained from the U.S. Geological Survey [14] as described previously [15]. We calculated the percentage of the population using well water by subtracting the percentage using public water from 100.

#### 2.2.5. Sulfuric Acid

Data on sulfuric acid releases for the year 1994 were obtained from the Toxics Release Inventory (TRI). Briefly, the TRI is a database of releases of chemicals that may pose a threat to human health and the environment [16]. Facilities that manufacture, use, or process these chemicals are required to report their releases to the Environmental Protection Agency. The data for sulfuric acid represents the total sulfuric acid release in pounds, including aerosol, land, and water releases.

#### 2.2.6. Ultraviolet Index

Several studies reported that low levels of exposure to sunlight may increase PD risk [17]. We examined sunlight using a validated measure of ultraviolet radiation, the UV Index [18].

#### 2.2.7. Smoking

Smoking is well-known to be inversely associated with PD. We included data on the prevalence of smoking to evaluate potential confounding by this variable. Data on smoking prevalence by state were obtained from the CDC [19].

### 2.3. Statistical Analysis

PD prevalence was modeled using linear regression. To determine the best fit model, model selection was run with PD prevalence as the outcome and each predictor variable separately. We generated a correlation matrix for all predictor variables and performed post hoc tests for collinearity. Only a single variable was retained among sets of highly inter-correlated predictor variables by comparing adjusted *R*^2^ values. Akaike information criterion (AIC) scores were used to select variables for a multivariable model. The AIC measures model quality compared with similar models by estimating goodness-of-fit penalized by the number of estimated parameters.

PD may have a subclinical period of decades [20]. Thus, individuals may be exposed to some etiologic factor(s) early life but be diagnosed at older ages, after they have migrated. We therefore repeated the analyses after censoring the data from Florida and Arizona, the states with the greatest number of migrant retirees.

## 3. Results

The mean, standard deviation, range, and locations for the highs and lows for the predictor variables are shown in Table 1. Briefly, the mean age-, race-, and sex-adjusted PD prevalence rate (calculated by Mantri et al. [11]), was 1325.0 (SD = 185.4) and ranged from 802.9 in Minnesota to 1719.8 in New York. The pH of precipitation ranged from 4.22 in Ohio to 5.43 in South Dakota (mean = 4.81, SD = 0.4). pH values were not available for four states (Connecticut, Delaware, Hawaii, and Rhode Island). The mean state-level precipitation was 37.5 inches (SD = 13.6) and ranged from 9.46 in Nevada to 59.15 in Louisiana. The API ranged nearly 10-fold, from 15.23 in Nevada to 131.6 in Alabama. Because the linearity assumption for the regression of sulfuric acid and PD prevalence was not met, the sulfuric acid data were log-transformed prior to analysis.

In univariate models, mean precipitation, the UV Index, and log sulfuric acid showed significant positive relationships with PD prevalence (*p* = 0.0001, *p* = 0.0249, *p* = 0.0381, respectively). Similarly, the API was significantly positively correlated with PD prevalence (β = 2.7, *t* = 4.48, *p* < 0.0001). Mean pH showed a significant negative correlation (i.e., the more acidic the precipitation, the higher the PD prevalence; *p* = 0.0002). Well water use was negatively correlated with PD prevalence (*p* = 0.0193). Because well water use was calculated as the difference between municipal water use and 100, this indicates that PD was positively correlated with the use of municipal water. The prevalence of smoking was not significantly associated with PD prevalence rates.

**Figure 1:**
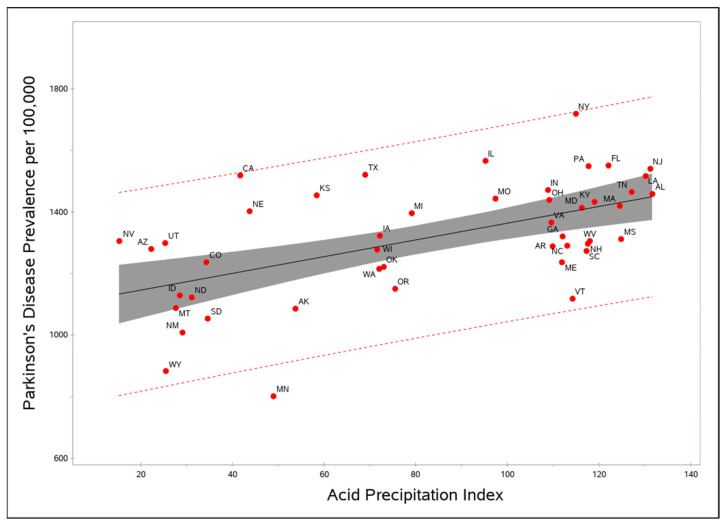
State-level prevalence of Parkinson’s disease vs. the quantity of acid rain, measured by the Acid Precipitation Index. Confidence limits of the mean and dotted lines are 95% confidence intervals.

Mean pH and mean precipitation were highly correlated with the API (*r* > 0.8). Because the API had a higher adjusted *R*^2^ than did mean pH and mean precipitation, it was retained in the model, and pH and precipitation were removed. In the final model, PD prevalence was significantly related to the API and (inversely) to well-water use (*p* < 0.001). Sulfuric acid releases stayed in the model because they significantly improved the model fit (Table 2). Analysis of the data after censoring data for Florida and Arizona produced results that were virtually unchanged. Figure 1 shows the scatter plot of PD prevalence and the API.

## 4. Discussion

In this ecologic study, we utilized published data on age-, race-, and sex-adjusted PD prevalence rates for U.S. states to examine suspected environmental risk factors for PD [11]. Our most important finding is that in multivariate analyses, PD prevalence rates were positively correlated with the quantity of acid precipitation (“acid rain”) (*p* < 0.0001). To our knowledge, this is the first study to report an association between PD and acid rain.

The state level prevalence of PD was positively correlated with the API, with industrial releases of sulfuric acid, and (inversely), with well-water use. Conversely, ultraviolet light was not significant when adjusted for other factors. The prevalence of smoking was not an important confounder in these relationships.

We observed a negative correlation between PD prevalence and well-water use. Well-water use has been reported to be positively associated with PD in many case-control studies. The literature also contains reports of null findings as well as protective effects for well water (see [21,22] for reviews). The majority of these studies were clinic-based and are susceptible to bias caused by the greater likelihood of cases to travel longer distances (i.e., from rural locations where wells are more common) to obtain care [23]. The inverse correlation that we observed is consistent with the largest of these studies, performed by Silver et al. [23]. These authors conducted a population-based case-control study among Medicare beneficiaries that included 89,790 incident PD cases and nearly 22,000,000 controls. They reported a robustly protective effect for well-water use (OR = 0.87, 95% C.I. = 0.85–0.89) with little evidence of increased risk from well water in any state. The inverse correlation we observed could reflect some (unknown) contaminant that is more common in municipal water. Alternately, as suggested by Silver et al., this finding may be a surrogate marker for other exposures that are more common in urban environments, e.g., pollution, that are associated with an increased risk for PD.

Each of our predictor variables that are characterized by acidity—the pH of precipitation, the quantity of precipitation, the API, and emissions of sulfuric acid—were significantly correlated with PD in univariate models. We note that even in non-polluted areas, “normal” rain has a pH of 5.0–5.5 and is thus acidic, a result of the atmospheric combination of moisture with carbon dioxide [24]. The fit of our final model was significantly improved by the inclusion of data on releases of sulfuric acid, which is a major component of air pollution and acid precipitation [25].

How can we understand the robust correlation of acid precipitation with PD prevalence? A plausible explanation involves the effects of acid rain on the aqueous concentration of metals. Metals, particularly lead, have been implicated repeatedly in Parkinson’s disease [26,27]. Acid rain is well-known to leach metals from watersheds and from plumbing into drinking water [28,29]. For example, the lead concentration of water that contains lead more than doubles as the pH drops from 7 to 6 [30]. This phenomenon is potentially important because lead is a major component of many water service lines, i.e., the underground pipes that connect homes to the municipal water main. It is noteworthy that the majority of the 6.1 million lead service lines in use in the U.S. are located in the Midwest [31].

**Figure 2:**
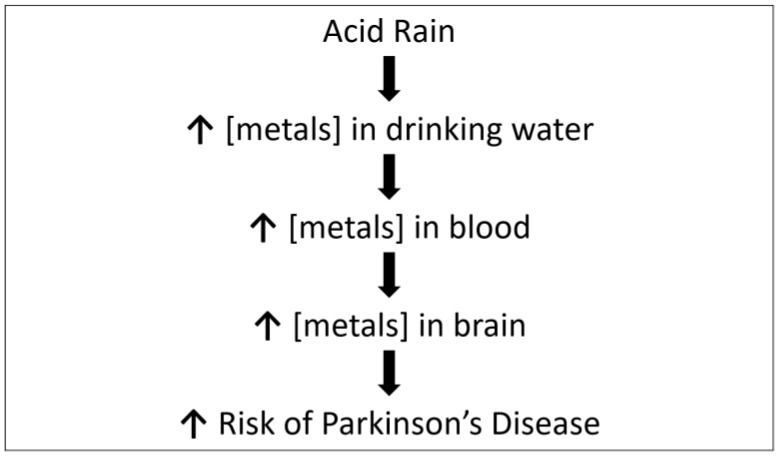
Hypothesized biological model of the acid rain hypothesis, “[]” indicates concentration.

For many metals, their concentrations in blood and in drinking water are highly correlated. For example, lead levels in blood increase as the cube root of lead levels in water [32]. Several reports indicate an increased body burden of lead in PD patients [33,34]. In animal models, lead reduces dopamine synthesis and promotes the accumulation of synuclein (reviewed in [5]). The aqueous concentration of other metals, e.g., iron, is also increased by acid rain, and these metals also may accumulate in the brain [5,35,36]. The acid rain hypothesis is summarized schematically in Figure 2.

Our study has several limitations. First, we used age-, race-, and sex-adjusted prevalence data for our measure of PD frequency. Because prevalence is influenced by disease duration, these data might exaggerate the occurrence of PD in more affluent states where PD longevity is greater. Additionally, data at the level of the state may be insensitive to capture potentially important variability that occurs at a finer scale. Second, we used the API as the principal measure of acid precipitation. The validity of this index as a measure of the effects of acid precipitation is unknown. However, acid precipitation is strongly associated with PD since both the quantity of precipitation and its pH (the component variables of the API) significantly predicted PD prevalence separately. Thirdly, we had no data on potential confounders such as pesticide use. Inspection of pesticide use maps for 1994 (and other years) for the pesticides most strongly implicated in PD, paraquat and rotenone, do not greatly resemble those of PD prevalence [37,38]. Additionally, it is likely that many individuals were exposed to etiologic factors in one state but were diagnosed after they migrated to another. This is likely to bias any correlation toward the null. The most important limitation of this study is that these are findings at the ecologic (group) level. As such, they indicate that PD prevalence rates are higher in states with higher exposure to acid precipitation and not that individuals who experience these exposures are at increased risk. Finally, we emphasize that we made no measures of metals; rather, the acid rain hypothesis is an effort to interpret our findings in the context of the published literature.

A strength of our study is that, although a role for acid rain in the etiology of neurodegenerative diseases has often been suggested (e.g., [39,40]), that hypothesis has never been tested. To our knowledge, this is the first study to provide evidence that acid precipitation is associated with PD. A role for acid precipitation in PD is biologically plausible and provides a mechanism for the frequently reported elevations of metals in the blood and tissues of patients with PD who do not report occupational exposure to metals [41].

The acid rain hypothesis suggests novel analytic studies. For example, in a population that uses municipal water, PD cases might show a history of greater water use than controls, a hypothesis that is testable using tax records. Similarly, exposures such as the use of metal premise plumbing may be more prevalent among PD cases than controls. Additionally, the metal contents of water samples from the homes of cases and controls could be compared directly. One such study has been reported (in Abstract), but the sample size (7 case-control pairs) is too small to permit meaningful conclusions [42,43].

## 5. Conclusions

This ecologic study of PD prevalence in U.S. states showed a positive association between PD and the quantity and the acidity of precipitation per state. Because acid precipitation is known to increase the concentration of metals in drinking water, these findings add support for a potential role of metals in the etiology of PD.

## Figures and Tables

**Table 1:** Summary statistics for predictor variables.

Variable	Mean (SD)	Range (State)
Parkinson’s disease prevalence rates	1325.0 (185.4)	802.9 (MN)–1719.8 (NY)
Sulfuric acid release amount	846,954.5 (1,299,945.3)	250 (SD)–7,609,755 (TN)
pH value	4.81 (0.4)	5.43 (SD)–4.22 (OH)
Precipitation amount	37.5 (13.6)	9.46 (NV)–59.15 (LA)
Acid Precipiation Index (API)	83.12 (38.5)	15.23 (NV)–131.6 (AL)
Well-water usage rate	17.3% (9.9)	3% (UT)–44% (ME)
Smoking prevalence rate	22.9% (3.0)	12.9% (UT)–30.5% (KY)
UV Index	25.1 (6.3)	14.63 (WA)–39.72 (FL)

**Table 2:** Regression models results for predicting PD prevalence.

Model	Predictor	β-Coefficient	*t*-Value	*p*-Value	AIC ^1^
pH alone	Mean pH	−255.7	−4.01	0.0002	469.90
Precipitation alone	Mean Precipitation	7.0	4.14	0.0001	509.95
Well water alone	Well-water use	−6.2	−2.42	0.0193	519.48
UV alone	UV Index	9.6	2.32	0.0249	499.23
Smoking alone	Smoking prevalence	6.0	0.69	0.4965	524.75
Sulfuric acid alone	Log sulfuric acid	24.3	2.13	0.0381	520.71
API alone	API	2.7	4.48	<0.0001	466.95
Full (API + well water + UV + smoking + sulfuric acid)	API	2.6	3.9	0.0004	447.85
	Well-water use	−7.7	−2.8	0.0078	
	UV Index	1.9	0.5	0.6532	
	Smoking prevalence	0.01	0	0.9989	
	Log sulfuric acid	15.8	1.3	0.1996	
Final (API + well water + sulfuric acid)	API	2.6	4.43	<0.0001	452.79
	Well-water use	−8.4		0.0001	
	Log sulfuric acid	18.0		0.0968	

^1^ AIC scores used in model selection with PROC GLMSELECT differed from the output of PROC REG for the models. AKI = Akaike information criterion; API = Acid Precipitation Index; UV = Ultraviolet.

## Data Availability

The datasets generated for this study are available from the corresponding author on reasonable request.
